# A Rare Case of Bilateral Cubital Valgus Secondary to Congenital Anterior Radial Head Dislocation

**DOI:** 10.7759/cureus.70855

**Published:** 2024-10-04

**Authors:** Pei Ern Ngo, Felix Lau Huey Yih, Satriya Sabir Husin Athar

**Affiliations:** 1 Orthopedic and Traumatology, National University Hospital Malaysia, Kuala Lumpur, MYS; 2 Orthopedics, Hospital Tuanku Ampuan Rahimah, Klang, Selangor, MYS

**Keywords:** anterior radial head dislocation, bilateral elbow deformity, congenital radial head dislocation, cubital valgus, hypoplastic capitulum, pseudoarthrosis

## Abstract

Congenital radial head dislocation (CRHD) is a rare orthopedic condition that frequently goes undiagnosed due to its asymptomatic presentation in early life. It is often identified incidentally during imaging studies for unrelated complaints or trauma. CRHD is often associated with inherited syndromes, emphasizing the genetic predisposition in this condition. While the majority of individuals remain asymptomatic in childhood, many may develop symptoms later in life, including joint stiffness, restricted range of motion, locking of the elbow joint, and aesthetic concerns related to elbow deformity. These delayed presentations can pose diagnostic challenges and contribute to a late diagnosis.

We report the case of a previously asymptomatic patient who presented after a fall with pain in the right elbow. Radiographic imaging revealed radial head dislocation, with characteristic findings including a dysplastic joint, hypoplastic capitulum, convex radial head, and pseudoarthrosis. Further evaluation with X-rays of the contralateral elbow showed similar abnormalities, leading to a diagnosis of bilateral congenital radial head dislocation.

Despite the structural abnormalities observed, the patient was managed conservatively without surgical intervention. Follow-up over a five-year period demonstrated satisfactory functional outcomes, with no significant worsening of symptoms or range of motion. This case highlights the importance of recognizing CRHD, particularly in patients who remain asymptomatic until adulthood or present following trauma. Conservative management can be an effective treatment approach in cases without severe functional impairment or pain, reinforcing the need for individualized treatment plans based on the patient's clinical presentation and functional demands.

## Introduction

Radial head dislocation may be a result of trauma or, less commonly, congenital. The estimated incidence of CRHD ranges from 0.06% to 0.16% [1-3]. The condition often goes unnoticed due to the absence of symptoms and typically becomes evident during adolescence when a noticeable deformity appears at the elbow joint [4]. Congenital radial head dislocation usually affects both elbows, though there have been a few instances of it occurring unilaterally. The progressive development of cubitus valgus, a condition where the forearm angles away from the body, can also occur and is attributed to the premature closure of the lateral humeral physis or the stunted growth of the lateral humeral condyle resulting from chronic radial head dislocation.

Many patients with unilateral or bilateral congenital radial head dislocation have associated anomalies, syndromes, or a family history of the condition [3]. Nevertheless, isolated cases of congenital radial head dislocation have also been reported [3-5]. Since it is typically asymptomatic or associated with mild symptoms that do not interfere with daily activities, treatment is generally conservative. Surgery is usually reserved for cases where patients experience persistent pain, restricted range of motion, or seek treatment for cosmetic reasons.

## Case presentation

**Figure 1:**
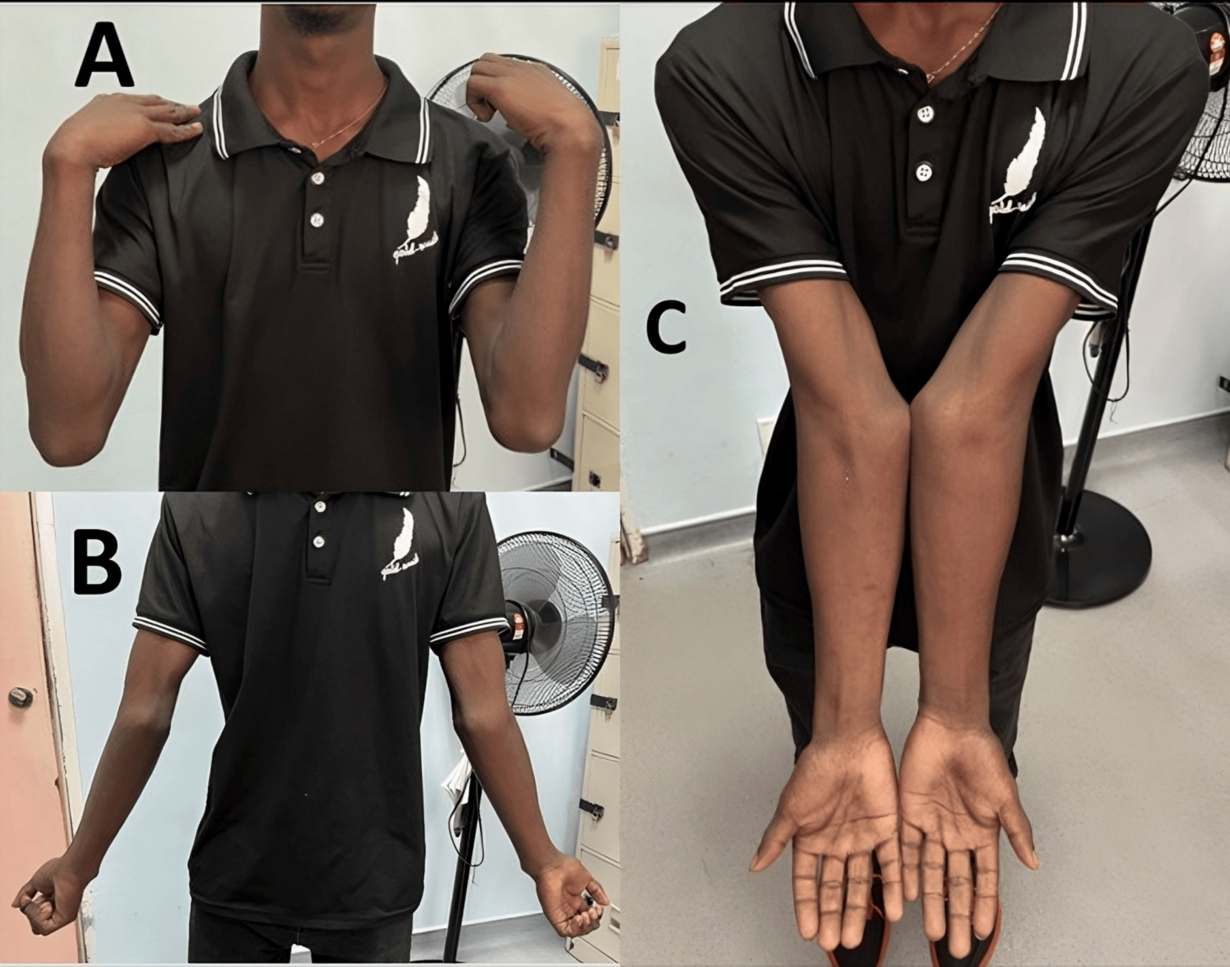
Full flexion (A) and extension (B) of bilateral elbow. Bilateral cubital valgus (C)

A 16-year-old boy with no known medical conditions presented to the emergency department with a history of slips and falls, landing on his right elbow. He was seen by the emergency team and referred to the orthopedic department for a right radial head dislocation. Upon assessment by the orthopedic team, a physical examination revealed tenderness over the right elbow with no visible wounds. He was found to have bilateral valgus deformity (Figure 1). A neurovascular examination of both upper limbs was unremarkable. The range of motion of the bilateral elbow was between 0 and 130 degrees, and he was able to achieve a full range of movement on pronation and supination bilaterally. 

**Figure 2:**
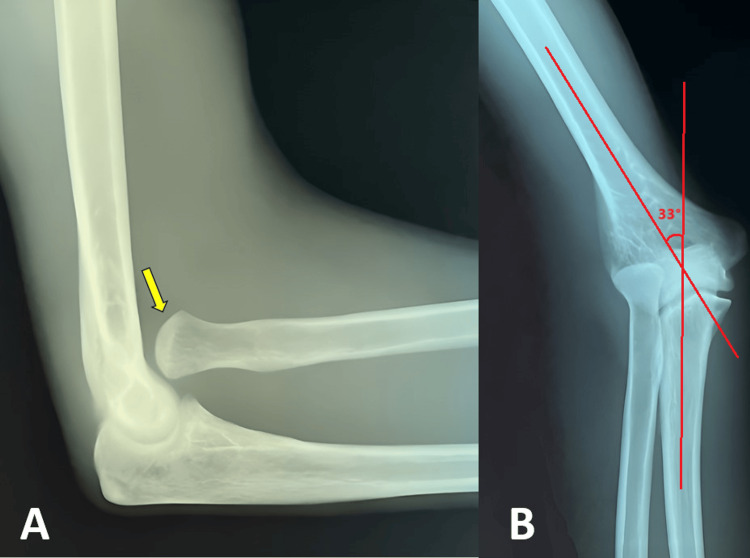
Lateral (A) and anteroposterior (B) radiological images of right elbow Yellow arrow showing a dislocated convex radial head

A general examination of other systems was grossly normal, with no features suggestive of syndromic conditions. A further review of the patient’s history revealed that both parents are non-consanguineous, with no family history of known inherited conditions or any family members with a similar presentation. He noticed prominent swelling over the medial aspect of both elbows at the age of 12; however, he did not seek medical attention as it did not affect his daily activities. A lateral radiograph of the elbow showed a convex radial head, and the radiocapitellar line did not intersect the capitellum, suggesting anterior dislocation of the radial head (Figure 2). An anteroposterior radiograph revealed a valgus deformity with a carrying angle of 33 degrees and flattening of the capitellum.

**Figure 3:**
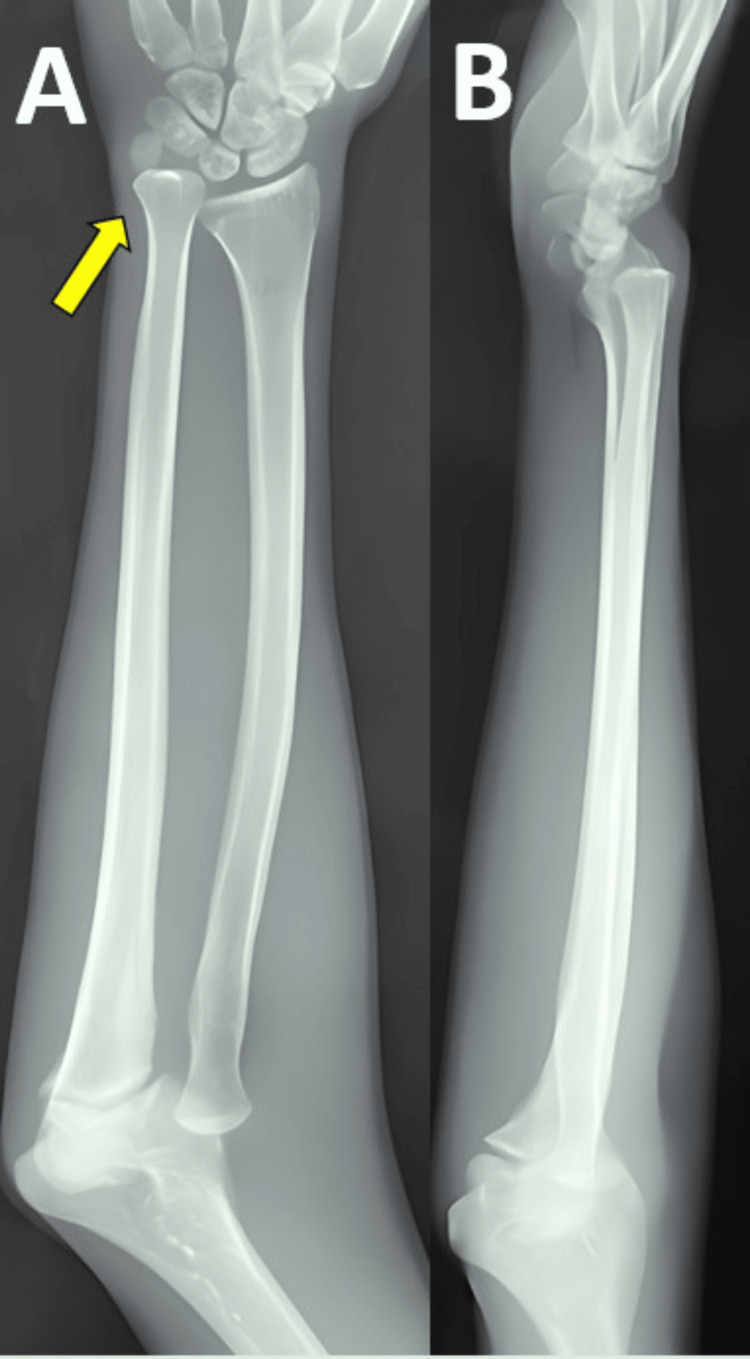
Anteroposterior (A) and lateral (B) radiological images of the right radius/ulna Yellow arrow showing ulnar variance of 2+ at the wrist joint

Right radius/ulna radiological images exhibit an ulnar variance of 2+ at the wrist joint (Figure 3). 

**Figure 4:**
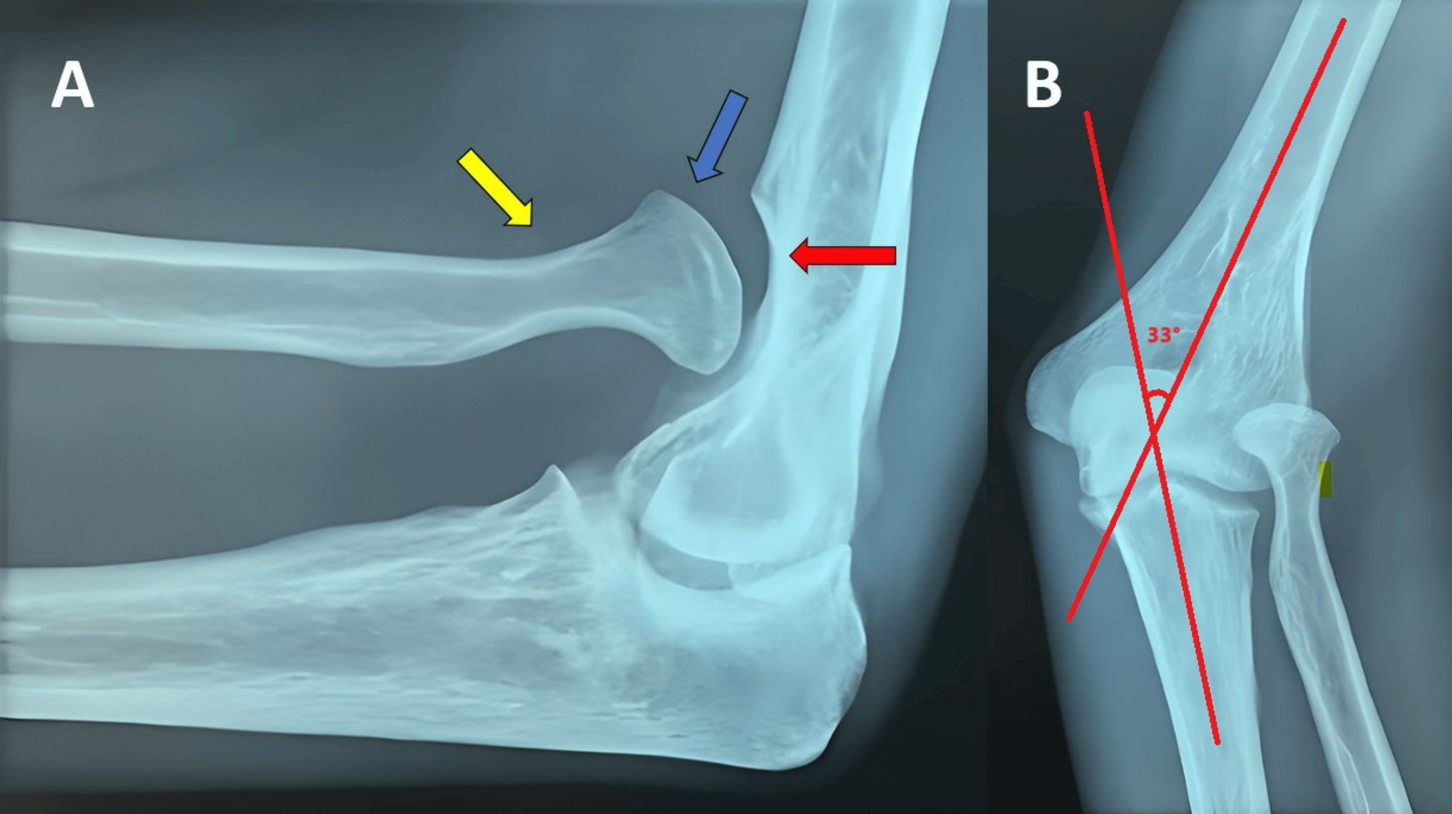
Lateral (A) and anteroposterior (B) radiological images of left elbow Yellow arrow showing a long and narrowed radial neck, blue arrow displaying a dome-shaped radial head, and red arrow exhibiting pseudoarthrosis over distal humerus

A contralateral radiograph of the elbow was done after assessment, which showed similar but more profound changes with pseudoarthrosis, a dome-shaped radial head with a long, narrow neck on the lateral view, and a carrying angle of 33 degrees from the anteroposterior view of the elbow (Figure 4). 

**Figure 5:**
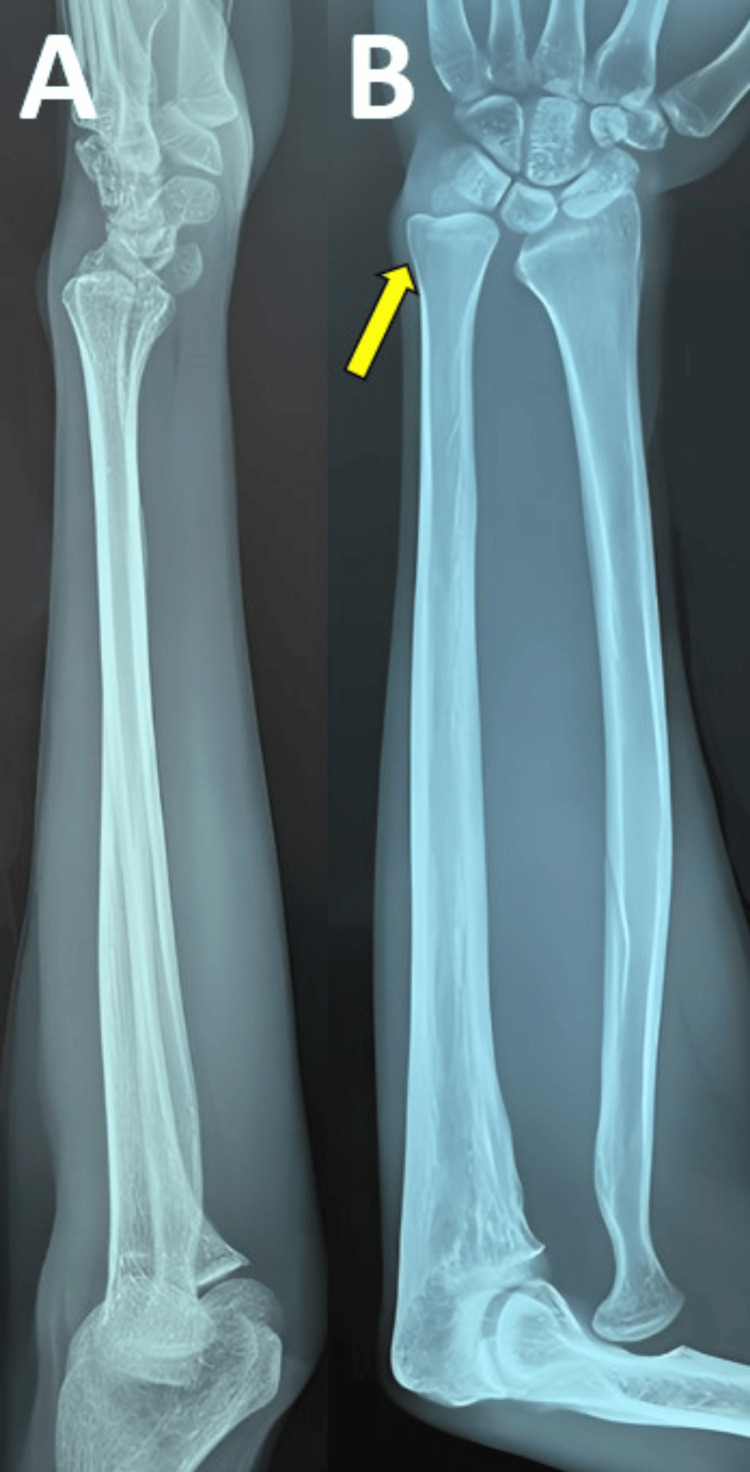
Lateral (A) and anteroposterior (B) radiological images of the left radius/ulna Yellow arrowing shows an ulnar variance of 2+.

The left radius/ulna radiological image shows an ulnar variance of 2+ over the wrist joint similar to the right side (Figure 5). 

He was diagnosed with bilateral congenital radial head dislocation (CRHD) and soft tissue injury of the right elbow and given analgesia. His symptoms improved significantly after subsequent follow-ups with no pain or limitation in the range of movements; he was treated conservatively with clinical observation. During a follow-up for five years, there was no progressive worsening of valgus deformity, and it remains asymptomatic.

## Discussion

The exact cause of congenital radial head dislocation (CRHD) is not well understood. However, it is believed to be the result of abnormal development of the elbow joint during fetal gestation and may be associated with genetic factors or occur spontaneously [1]. Posterior radial head dislocation remains the most common, accounting for 65 percent of cases, while anterior and lateral dislocations occur in 18% and 17% of cases, respectively [2]. In our case of anterior dislocation, overgrowth of the proximal radius might have resulted in increased pressure or repetitive minor trauma on the lateral physis, causing growth arrest. As a result of the continuing growth of the medial epiphysis, progressive cubital valgus developed [4-6].

CRHD is associated with other conditions in about one-third of the cases [7]. Upper extremity anomalies associated with this condition include ulnar ray defects, radial ray defects, congenital radioulnar synostosis, Madelung's deformity, and terminal hemimelia. Lower extremity anomalies associated with this condition include developmental dysplasia of the hip, clubfoot, and tibiofibular synostosis. Generalized conditions associated with radial head dislocation include Cornelia de Lange syndrome, Ehlers-Danlos syndrome, Klippel-Feil syndrome, nail-patellar syndrome, Klinefelter's syndrome, arthrogryposis, and Silver's syndrome. In isolated cases, the condition is believed to be autosomal dominant, and there have been reported cases in consanguineous marriages.

Symptoms of congenital radial head dislocation can vary in severity [1,3,8]. In mild cases, the symptoms may be subtle, and the condition may go unnoticed until later, as seen in our case. In more severe cases, it can present with deformity, pain, or a reduction in the range of movement at the elbow joint, leading to functional limitation.

Diagnosis of CRHD typically involves a combination of clinical examination and imaging studies [1,3,9]. CRHD can be distinguished from traumatic dislocation through detailed history-taking and thorough examination. With a high index of suspicion, radiological images are done to confirm the diagnosis. McFarland describes radiological features indicative of congenital radial head dislocation as the absence or hypoplasia of the capitellum, relative shortening of the ulna or overlength of the radius, prominent ulnar epicondyle, grooving of the distal radius, a partially defective trochlea, and a dome-shaped radial head with a long, narrow neck [9].

As symptoms are often mild and do not significantly affect a patient’s function, observation alone is the standard treatment with a good functional outcome [10, 11]. Surgical management is usually indicated in cases of limitation in the range of movement, persistent pain, or for cosmetic reasons. Surgical options include early reconstruction or delayed intervention in skeletally mature patients. Described surgical interventions include open reduction of the radiocapitellar joint with annular ligament reconstruction, ulnar osteotomy, radial head excision, and elbow arthroplasty [1,3,11]. Radial head excision, described in various literature for the treatment of CRHD, remains the treatment of choice due to its relative simplicity and fewer complications [7]. It is important to note that while radial head excision relieves pain and improves appearance, it does not increase motion.

## Conclusions

Congenital radial head dislocation (CRHD) is a condition that often remains unnoticed due to its asymptomatic nature. It is often diagnosed incidentally following a trauma. This case illustrates the importance of thorough history taking, examination, and maintaining an index of suspicion in patients with bilateral elbow deformities to avoid unnecessary intervention. Classical radiographical findings include anterior radial head dislocation, pseudoarthrosis, hypoplastic capitellum, and convex radial head. If a child has congenital radial head dislocation in one or both arms, it is important to investigate for other abnormalities or syndromes.

Conservative management with observation alone remains the first-line treatment for CRHD. Surgical intervention is reserved for patients with persistent pain, limited range of motion, or cosmetic concerns.
